# Supplementary material for: Neurological manifestations of scrub typhus infection: A systematic review and meta-analysis of clinical features and case fatality
Source: PLoS Negl Trop Dis. 2022 Nov 28;16(11):e0010952. doi: 10.1371/journal.pntd.0010952 (PMC9731453; doi:10.1371/journal.pntd.0010952)
Supplement: S1 Table — (DOCX) [file pntd.0010952.s001.docx]

**S1 Table – Search strategy**

Pubmed (MEDLINE) search strategy

| **Search** | **Query** | **Results** |
| --- | --- | --- |
| #5 | #3 AND #4 | 267 |
| #4 | **("2000/01/01"[Date - Publication] : "2022/04/22"[Date - Publication])** | 20501525 |
| #3 | #1 AND #2 | 308 |
| #2 | **(encephalitis OR encephal* OR meningoencephalitis OR Meningitis OR Central nervous system OR CNS)** | 1842406 |
| #1 | **(scrub typhus OR Orientia tsutsugamushi OR Rickettsia tsutsugamushi OR Orientia tsu OR Akamushi disease OR Japanese river fever OR mite typhus OR tropical typhus OR tsutsugamushi disease)** | 3194 |

Scopus search strategy

| **Search** | **Query** | **Results** |
| --- | --- | --- |
| #3 | #1 AND #2 | 447 |
| #2 | TITLE-ABS-KEY ("encephalitis" OR "encephal*" OR "meningoencephalitis" OR "Meningitis" OR "Central nervous system" OR "CNS" ) | 1842406 |
| #1 | TITLE-ABS-KEY ( "scrub typhus"  OR  "Orientia tsutsugamushi"  OR  "Rickettsia tsutsugamushi"  OR  "Orientia tsu"  OR  "Akamushi disease"  OR  "Japanese river fever"  OR  "mite typhus"  OR  "tropical typhus"  OR  "tsutsugamushi disease" ) | 3652 |
