# Supplementary material for: Neurological manifestations of scrub typhus infection: A systematic review and meta-analysis of clinical features and case fatality
Source: PLoS Negl Trop Dis. 2022 Nov 28;16(11):e0010952. doi: 10.1371/journal.pntd.0010952 (PMC9731453; doi:10.1371/journal.pntd.0010952)
Supplement: S2 Table — (DOCX) [file pntd.0010952.s002.docx]

**S2 Table – Inclusion criteria**

| **Include** | **Exclude** |
| --- | --- |
| All studies reporting outcomes in scrub typhus with neurological manifestations | Reviews, editorials, abstracts and case reports |
| Published after 01/01/2000 | No outcome reported |
| Confirmed scrub typhus diagnosis by serology | No demographics of cohort reported |
| Any treatment strategy | Clinical diagnosis of scrub typhus |
| Any patient size >10 | Case reports or case series <10 |
| English language | Co-infection with scrub typhus |
