# Supplementary material for: Neurological manifestations of scrub typhus infection: A systematic review and meta-analysis of clinical features and case fatality
Source: PLoS Negl Trop Dis. 2022 Nov 28;16(11):e0010952. doi: 10.1371/journal.pntd.0010952 (PMC9731453; doi:10.1371/journal.pntd.0010952)
Supplement: S3 Table — (DOCX) [file pntd.0010952.s003.docx]

**S3 Table – Data collected**

| Paper  Author + Year  Location  Setting  Study type  Duration of study  Duration of recruitment in months  Number of patients  Age median (IQR)  Age mean (SD)  Number of female  Number of deaths  No disability  Mild disability  Moderate disability  Severe disability  Dead after discharge  Encephalitis or Meningitis or both (AES - 0, Meningitis - 1, ME - 2)  Median GCS on admission  Median Symptom duration in days | Fever  Headache  Altered sensorium / GCS  Neck stiffness/nuchal rigidity  Seizures  Dyspnoea  Cough  Diarrhoea  Abdominal pain  Vomiting  Lymphadenopathy  Eschar  Rash  Organomegaly  Jaundice  Treated? (no 0, yes 1, unknown 2)  Azithromycin  Doxycycline  Rifampicin  Chloramphenicol  Combined |
| --- | --- |
