# Supplementary material for: Neurological manifestations of scrub typhus infection: A systematic review and meta-analysis of clinical features and case fatality
Source: PLoS Negl Trop Dis. 2022 Nov 28;16(11):e0010952. doi: 10.1371/journal.pntd.0010952 (PMC9731453; doi:10.1371/journal.pntd.0010952)
Supplement: S4 Table — (DOCX) [file pntd.0010952.s004.docx]

**S4 Table – Bias assessment**

| **Paper** | **Did the study address a clearly focused, relevant issue?** | **Was the cohort recruited in an acceptable way?** | **0 =poor, 1=fair, 2=good** | **Have the authors identified all important confounding factors?** | **Have they accounted for confounding factors in the design and/or analysis?** | **0 = poor, 1=fair, 2=good** | **Was the follow up of subjects complete enough? (Retrospective get 0)** | **Defined follow up?** | **0 =poor, 1=fair, 2=good** | **Do you believe the results?** | **Can the results be applied to   the local population?** | **Do the results of this study fit   with other available   evidence?** | **0,1 =poor, 2=fair, 3=good** | **Total**  **6-7 good,**  **4-5 moderate, 3 and below poor** |
| --- | --- | --- | --- | --- | --- | --- | --- | --- | --- | --- | --- | --- | --- | --- |
| Abhilash 2015 [1] | ✔ | ✔ | 2 | ✔ | ✔ | 2 | ✘ | ✘ | 0 | ✔ | ✔ | ✔ | 3 | 5 |
| Alam 2020 [2] | ✔ | ✔ | 2 | ✔ | ✔ | 2 | ✔ | ✔ | 2 | ✔ | ✘ | ✔ | 2 | 6 |
| Arora 2021 [3] | ✘ | ✔ | 1 | ✔ | ✔ | 2 | ✔ | ✔ | 2 | ✔ | ✘ | ✔ | 2 | 5 |
| Bhat 2016 [4] | ✔ | ✔ | 2 | ✔ | ✔ | 2 | ✔ | ✔ | 2 | ✔ | ✘ | ✔ | 2 | 6 |
| Bhat 2017 [5] | ✔ | ✔ | 2 | ✔ | ✔ | 2 | ✔ | ✔ | 2 | ✔ | ✘ | ✔ | 2 | 6 |
| Dittrich 2015 [6] | ✔ | ✔ | 2 | ✔ | ✔ | 2 | ✔ | ✘ | 1 | ✔ | ✔ | ✔ | 3 | 6 |
| Gangwar 2020 [7] | ✔ | ✔ | 2 | ✘ | ✘ | 0 | ✘ | ✔ | 1 | ✔ | ✘ | ✔ | 2 | 5 |
| Jamil 2015 [8] | ✔ | ✔ | 2 | ✔ | ✘ | 1 | ✘ | ✘ | 0 | ✔ | ✘ | ✘ | 1 | 3 |
| Kim 2013 [9] | ✔ | ✘ | 1 | ✘ | ✘ | 0 | ✘ | ✘ | 0 | ✔ | ✘ | ✔ | 2 | 3 |
| Dinesh Kumar 2018 [10] | ✔ | ✔ | 2 | ✘ | ✘ | 0 | ✔ | ✘ | 1 | ✔ | ✘ | ✔ | 2 | 5 |
| Lee 2017 [11] | ✔ | ✔ | 2 | ✘ | ✘ | 0 | ✘ | ✘ | 0 | ✔ | ✔ | ✔ | 3 | 5 |
| Misra 2014 [12] | ✔ | ✔ | 2 | ✔ | ✔ | 2 | ✔ | ✔ | 2 | ✔ | ✔ | ✔ | 3 | 7 |
| Mittal 2018 [13] | ✔ | ✘ | 1 | ✔ | ✔ | 2 | ✘ | ✘ | 0 | ✔ | ✘ | ✔ | 2 | 3 |
| Rana 2016 [14] | ✔ | ✔ | 2 | ✘ | ✘ | 0 | ✘ | ✘ | 0 | ✔ | ✘ | ✔ | 2 | 4 |
| Rose 2017 [15] | ✔ | ✔ | 2 | ✔ | ✔ | 2 | ✘ | ✘ | 0 | ✔ | ✘ | ✔ | 2 | 4 |
| Sharma 2015 [16] | ✔ | ✔ | 2 | ✘ | ✘ | 0 | ✘ | ✘ | 0 | ✔ | ✘ | ✔ | 2 | 4 |
| Thakur 2020 [17] | ✘ | ✔ | 1 | ✘ | ✔ | 1 | ✘ | ✘ | 0 | ✔ | ✔ | ✔ | 3 | 4 |
| Valappil 2017 [18] | ✔ | ✔ | 2 | ✔ | ✔ | 2 | ✘ | ✘ | 0 | ✔ | ✘ | ✔ | 2 | 4 |
| Viswanathan 2013 [19] | ✔ | ✔ | 2 | ✔ | ✔ | 2 | ✘ | ✘ | 0 | ✔ | ✘ | ✔ | 2 | 4 |

**References:**

1. Abhilash KP, Gunasekaran K, Mitra S, Patole S, Sathyendra S, Jasmine S, et al. Scrub typhus meningitis: An under-recognized cause of aseptic meningitis in India. Neurol India. 2015;63(2):209-14. Epub 2015/05/08. doi: 10.4103/0028-3886.156282. PubMed PMID: 25947985.

2. Alam A, Agarwal P, Prabha J, Jain A, Kalyan RK, Kumar C, et al. Prediction Rule for Scrub Typhus Meningoencephalitis in Children: Emerging Disease in North India. J Child Neurol. 2020;35(12):820-7. Epub 2020/06/26. doi: 10.1177/0883073820933148. PubMed PMID: 32580611.

3. Arora S, Abhilash KPP, Mitra S, Hazra D, Gunasekharan K, Yesudass P. Is cerebrospinal fluid lactate useful in differentiating scrub typhus meningitis from aseptic, bacterial and tuberculous meningitis? Trop Doct. 2021;51(1):64-71. Epub 2020/12/02. doi: 10.1177/0049475520975957. PubMed PMID: 33259753.

4. Bhat NK, Pandita N, Saini M, Dhar M, Ahmed S, Shirazi N, et al. Scrub Typhus: A Clinico-Laboratory Differentiation of Children with and without Meningitis. J Trop Pediatr. 2016;62(3):194-9. Epub 2016/02/07. doi: 10.1093/tropej/fmv097. PubMed PMID: 26851433.

5. Bhat NKA, V; Kakati, B.; Mittal, G.; Pandita, N.; Wasim, S.; Gupta, A.; Dhar, M.; Ahmed, S. Scrub Typhus Meningitis versus Acute Bacterial Meningitis—A Clinical-Laboratory Differential Diagnosis. J Pediatr Infect Dis. 2017;(12(02)):119-23. doi: 10.1055/s-0037-1601339.

6. Dittrich S, Rattanavong S, Lee SJ, Panyanivong P, Craig SB, Tulsiani SM, et al. Orientia, rickettsia, and leptospira pathogens as causes of CNS infections in Laos: a prospective study. Lancet Glob Health. 2015;3(2):e104-12. Epub 2015/01/27. doi: 10.1016/S2214-109X(14)70289-X. PubMed PMID: 25617190; PubMed Central PMCID: PMCPMC4547322.

7. Prakash Gangwar S, Thangaraj JWV, Zaman K, Vairamani V, Mittal M, Murhekar M. Sequelae Following Acute Encephalitis Syndrome Caused by Orientia Tsutsugamushi. Pediatr Infect Dis J. 2020;39(5):e52-e4. Epub 2020/04/18. doi: 10.1097/INF.0000000000002595. PubMed PMID: 32301923.

8. Jamil MD, Hussain M, Lyngdoh M, Sharma S, Barman B, Bhattacharya PK. Scrub typhus meningoencephalitis, a diagnostic challenge for clinicians: A hospital based study from North-East India. J Neurosci Rural Pract. 2015;6(4):488-93. Epub 2016/01/12. doi: 10.4103/0976-3147.169769. PubMed PMID: 26752890; PubMed Central PMCID: PMCPMC4692003.

9. Kim DM, Chung JH, Yun NR, Kim SW, Lee JY, Han MA, et al. Scrub typhus meningitis or meningoencephalitis. Am J Trop Med Hyg. 2013;89(6):1206-11. Epub 2013/10/30. doi: 10.4269/ajtmh.13-0224. PubMed PMID: 24166036; PubMed Central PMCID: PMCPMC3854902.

10. Dinesh Kumar N, Arun Babu T, Vijayadevagaran V, Ananthakrishnan S, Kittu D. Clinical Profile of Scrub Typhus Meningoencephalitis among South Indian Children. J Trop Pediatr. 2018;64(6):472-8. Epub 2017/12/23. doi: 10.1093/tropej/fmx096. PubMed PMID: 29272545.

11. Lee HS, Sunwoo JS, Ahn SJ, Moon J, Lim JA, Jun JS, et al. Central Nervous System Infection Associated with Orientia tsutsugamushi in South Korea. Am J Trop Med Hyg. 2017;97(4):1094-8. Epub 2017/08/19. doi: 10.4269/ajtmh.17-0077. PubMed PMID: 28820719; PubMed Central PMCID: PMCPMC5637598.

12. Misra UK, Kalita J, Mani VE. Neurological manifestations of scrub typhus. J Neurol Neurosurg Psychiatry. 2015;86(7):761-6. Epub 2014/09/12. doi: 10.1136/jnnp-2014-308722. PubMed PMID: 25209416.

13. Mittal M, Bondre V, Murhekar M, Deval H, Rose W, Verghese VP, et al. Acute Encephalitis Syndrome in Gorakhpur, Uttar Pradesh, 2016: Clinical and Laboratory Findings. Pediatr Infect Dis J. 2018;37(11):1101-6. Epub 2018/05/11. doi: 10.1097/INF.0000000000002099. PubMed PMID: 29746378.

14. Rana A, Mahajan SK, Sharma A, Sharma S, Verma BS, Sharma A. Neurological manifestations of scrub typhus in adults. Trop Doct. 2017;47(1):22-5. Epub 2016/04/10. doi: 10.1177/0049475516636543. PubMed PMID: 27059055.

15. Rose W, Ghosh U, Punnen A, Sarkar R, Prakash JJA, Verghese VP. Comparison of Scrub Typhus With and Without Meningitis. Indian J Pediatr. 2017;84(11):833-7. Epub 2017/07/05. doi: 10.1007/s12098-017-2403-4. PubMed PMID: 28674823.

16. Sharma SR, Masaraf H, Lynrah KG, Lyngdoh M. Tsutsugamushi Disease (Scrub Typhus) Meningoencephalitis in North Eastern India: A Prospective Study. Ann Med Health Sci Res. 2015;5(3):163-7. Epub 2015/06/23. doi: 10.4103/2141-9248.157486. PubMed PMID: 26097756; PubMed Central PMCID: PMCPMC4455004.

17. Thakur CK, Chaudhry R, Gupta N, Vinayaraj EV, Singh V, Das BK, et al. Scrub typhus in patients with acute febrile illness: a 5-year study from India. QJM. 2020;113(6):404-10. Epub 2019/12/04. doi: 10.1093/qjmed/hcz308. PubMed PMID: 31790119.

18. Valappil AV, Thiruvoth S, Peedikayil JM, Raghunath P, Thekkedath M. Differential diagnosis of scrub typhus meningitis from tuberculous meningitis using clinical and laboratory features. Clin Neurol Neurosurg. 2017;163:76-80. Epub 2017/10/28. doi: 10.1016/j.clineuro.2017.10.022. PubMed PMID: 29078126.

19. Viswanathan S, Muthu V, Iqbal N, Remalayam B, George T. Scrub typhus meningitis in South India--a retrospective study. PLoS One. 2013;8(6):e66595. Epub 2013/06/27. doi: 10.1371/journal.pone.0066595. PubMed PMID: 23799119; PubMed Central PMCID: PMCPMC3682970.
