# Supplementary material for: Neurological manifestations of scrub typhus infection: A systematic review and meta-analysis of clinical features and case fatality
Source: PLoS Negl Trop Dis. 2022 Nov 28;16(11):e0010952. doi: 10.1371/journal.pntd.0010952 (PMC9731453; doi:10.1371/journal.pntd.0010952)
Supplement: S5 Table — (DOCX) [file pntd.0010952.s005.docx]

**S5 Table – Sensitivity analysis (excluding studies which used Weil-Felix test in diagnosis)**

|  | **All studies** | | | **Excluding studies using WFT** | | |
| --- | --- | --- | --- | --- | --- | --- |
|  | **CFR or WPP** | **95% CI** | **I^2^** | **CFR or WPP** | **95% CI** | **I^2^** |
| **Case fatality ratio** | 3.64 | 1.52 – 6.38 | 67.4% | 5.33 | 2.89 – 8.32 | 59.5% |
| **Fever (%)** | 100.0% | 99.5% - 100.0% | 47.8% | 100.0% | 98.9% - 100.0% | 59.6% |
| **Headache (%)** | 65.0% | 51.5% - 77.6% | 95.1% | 58.2% | 43.1% - 72.6% | 95.3% |
| **Altered sensorium (%)** | 67.4% | 54.9% - 78.8% | 93.3% | 63.0% | 49.3% - 75.8% | 93.8% |
| **Neck stiffness (%)** | 55.6% | 29.4% - 80.4% | 96.3% | 53.8% | 23.3% - 82.9% | 97.0% |
| **Seizures (%)** | 43.8% | 25.1% - 63.4% | 96.8% | 47.1% | 25.8% - 69.0% | 97.2% |
| **Dyspnoea (%)** | 25.2% | 10.3% - 43.6% | 94.1% | 23.4% | 8.2% - 43.1% | 92.3% |
| **Cough (%)** | 26.2% | 12.6% - 42.3% | 88.7% | 28.2% | 10.0% - 50.8% | 91.1% |
| **Diarrhoea (%)** | 5.8% | 3.4% - 8.6% | 35.9% | 6.2% | 3.4% - 9.6% | 48.7% |
| **Abdominal pain (%)** | 24.3% | 16.5% - 33.0% | 82.2% | 18.2% | 12.7% - 24.3% | 62.3% |
| **Vomiting (%)** | 46.2% | 34.1% - 58.5% | 91.6% | 37.9% | 25.4% - 51.1% | 91.4% |
| **Jaundice (%)** | 8.3% | 2.9% - 15.5% | 72.9% | 5.6% | 2.5% - 9.6% | 43.5% |
| **Organomegaly (%)** | 48.0% | 38.3% - 57.7% | 85.4% | 48.8% | 38.5 - 59.2% | 84.1% |
| **Lymphadenopathy (%)** | 24.1% | 11.8% - 38.9% | 87.8% | 18.0% | 10.2% - 27.2% | 66.5% |
| **Eschar (%)** | 20.8% | 9.8% - 34.3% | 95.4% | 19.2% | 7.4% - 34.6% | 96.1% |
| **Rash (%)** | 20.2% | 11.1% - 31.1% | 91.3% | 22.7% | 11.7% - 35.9% | 92.7% |
