# Supplementary figures and images for: Neurological manifestations of scrub typhus infection: A systematic review and meta-analysis of clinical features and case fatality
Source: PLoS Negl Trop Dis. 2022 Nov 28;16(11):e0010952. doi: 10.1371/journal.pntd.0010952 (PMC9731453; doi:10.1371/journal.pntd.0010952)

**S1 Fig – Funnel plots**

Paediatric cohorts

**
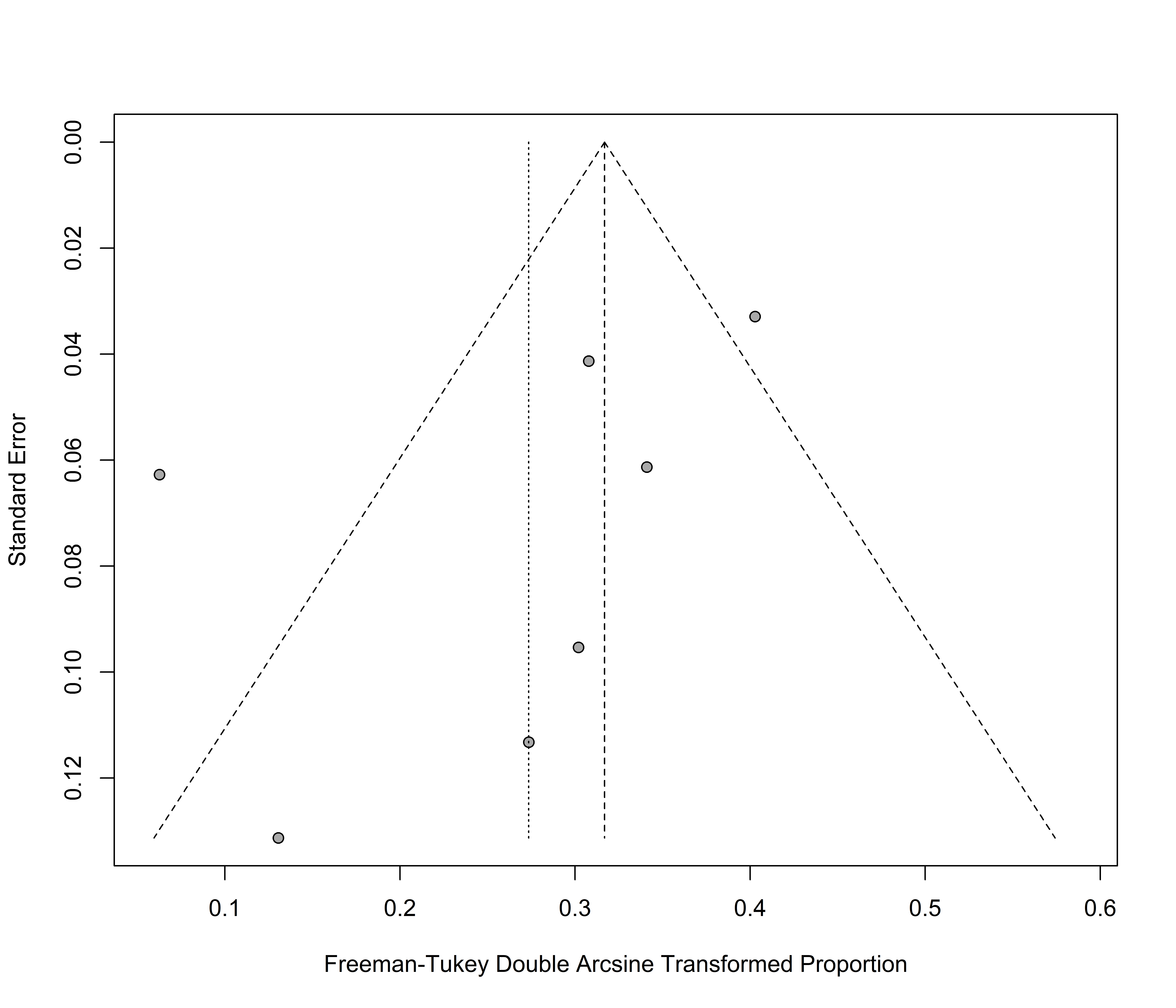
**

Adult cohorts

**
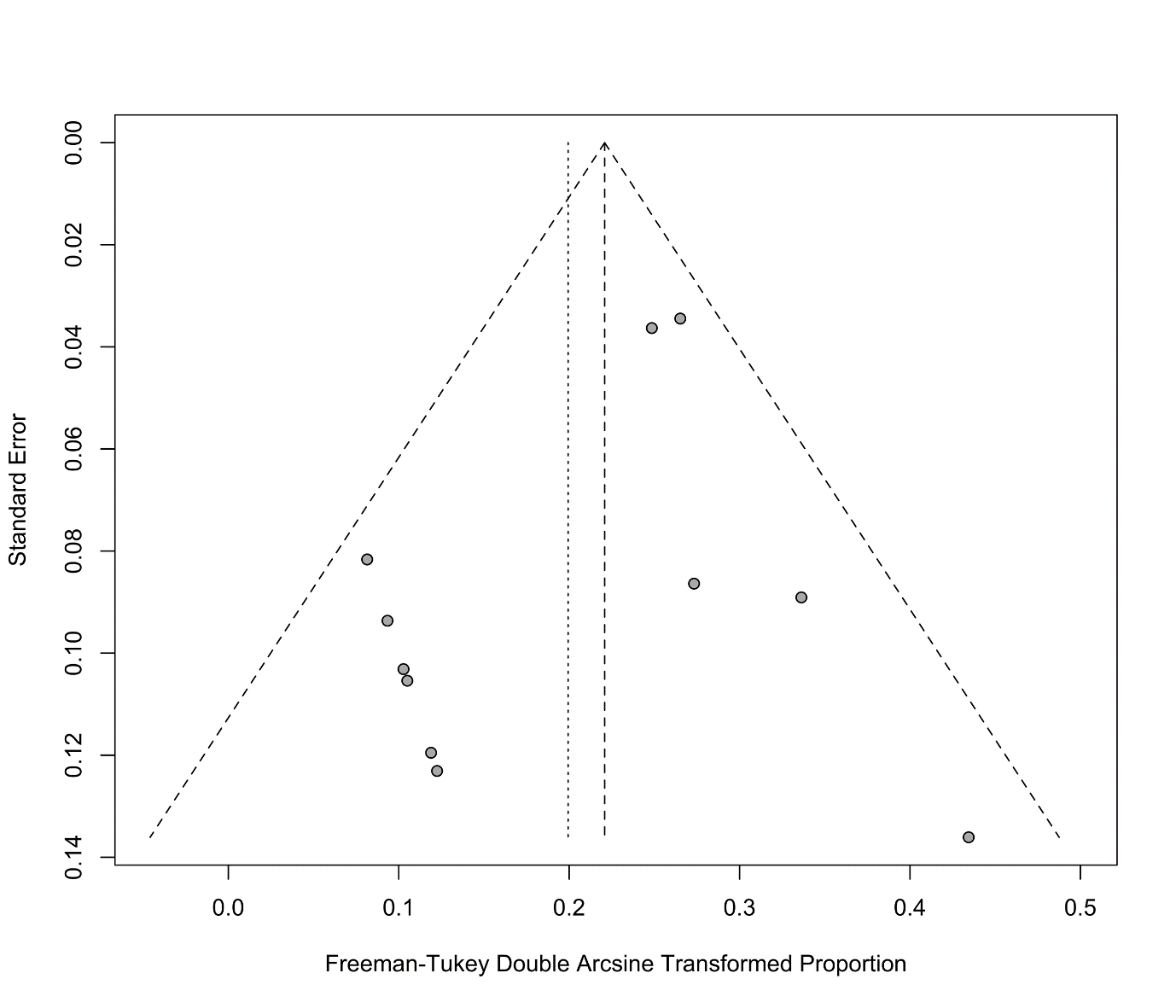
**

All cohorts


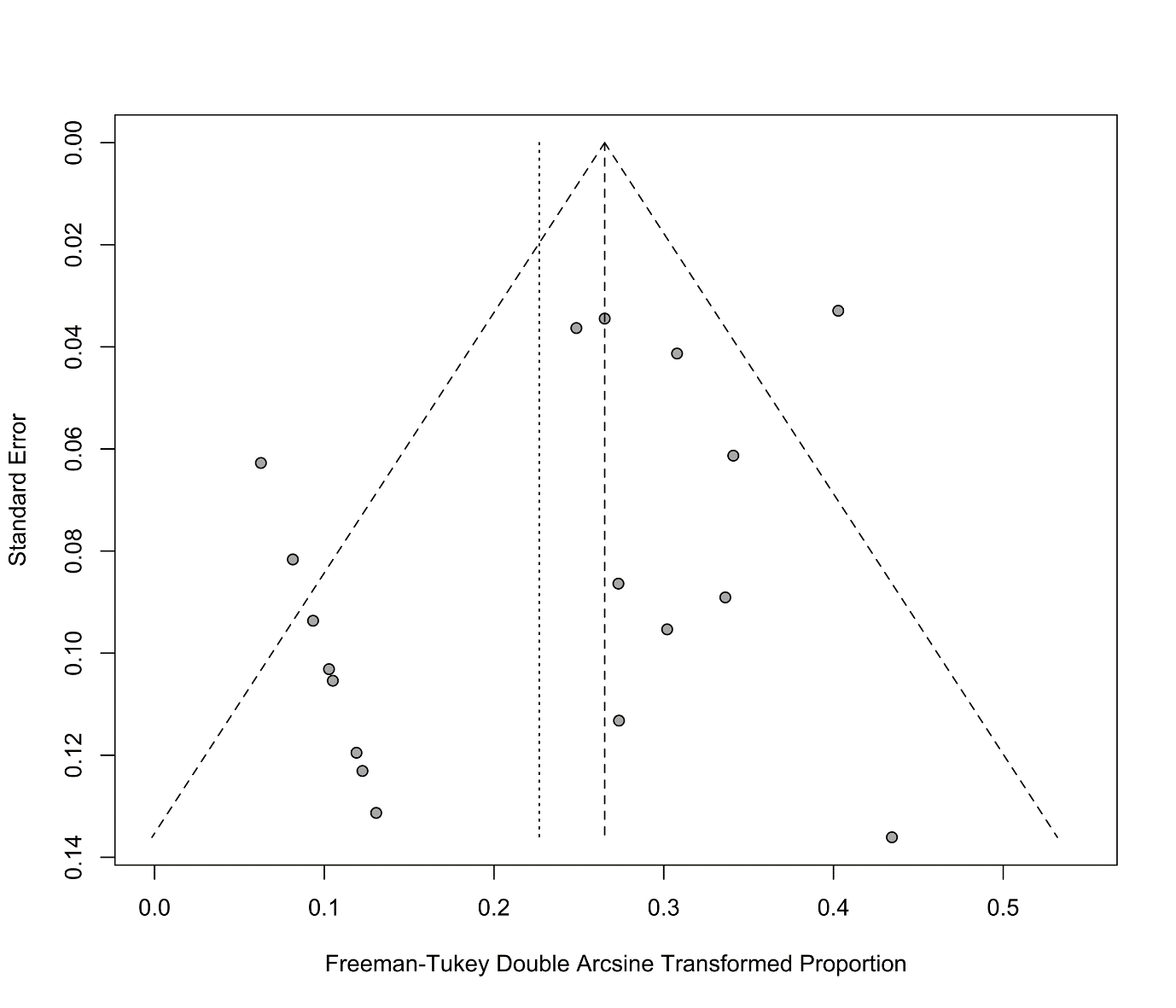

Supplement: S1 Fig — (DOCX) [file pntd.0010952.s006.docx]
