# Supplementary material for: Neurological manifestations of scrub typhus infection: A systematic review and meta-analysis of clinical features and case fatality
Source: PLoS Negl Trop Dis. 2022 Nov 28;16(11):e0010952. doi: 10.1371/journal.pntd.0010952 (PMC9731453; doi:10.1371/journal.pntd.0010952)
Supplement: Attachment — Submitted filename: Response to reviewers.pdf [file pntd.0010952.s007.pdf]

Thank you for taking time to review our manuscript and for identifying these points. We have been able to include all the suggested changes in the revised manuscript and have resubmitted this with the below point-by-point response.

Reviewer 1 comments:

1. Is lymphadenopathy a pathognomonic feature of scrub typhus? Please give references for this.

Response:

Thank you for highlighting this. We used the features of scrub typhus as stated in Manson's Tropical Infectious Diseases (DOI:10.1016/C2010-0-66223-7) which suggests that "The presenting features are typically fever, generalized or regional lymphadenopathy, a macular or maculopapular rash, severe headache and myalgia". We acknowledge that lymphadenopathy may not be considered 'pathognomonic' and so have amended our reference to a pathognomonic feature as only the eschar. We have amended the manuscript to highlight that lymphadenopathy may be considered a 'classical' feature of scrub typhus instead.

2. In para 4 of Introduction, authors mention inclusion of case series in their review but in para 2 of methods, they have mentioned excluding case series. This needs clarification.

Response:

Thank you for raising this point and sorry for this oversight on our behalf. In the revised manuscript we have corrected this – we excluded case series in our study.

3. Methodology: Scrub typhus diagnosed by IFA, Weil-Felix and ELISA have been included in these study for review. The specificity of Weil-Felix is poor and should ideally not be included in review such as these. The authors should take a look at their data after removing patients diagnosed solely by Weil-Felix test.

Response:

Thank you for asking us to clarify this. We have added a whole section regarding this point in our methodology. To ensure the validity of our meta-analyses, we conducted sensitivity analysis to see the robustness of our results when removing patients diagnosed by Weil-Felix test (WFT). This is illustrated in the appendix and is shown to cause no significant change in

our results. The reason why we still included studies using WFT is that many studies solely had access to this and that there is still utility of the WFT in corroboration with clinical features in such settings. We also added the drawbacks in using serological tests as a diagnostic tool in our limitations.

4. Results: Dyspnoea was present in 25.1% of patients. Was this ARDS? If yes, then it would be difficult to delineate in which patients mortality was due to CNS disease versus ARDS. The authors have not mentioned anything related to this.

Response:

Thank you for raising this interesting point. Sadly, it is hard to determine what the severity of the dyspnoea in the patients were – and whether ARDS leading to death was present. In our methodology it would be impossible to determine the cause of death in the patient cohort. We did not study the cause of mortality in patients – but instead to understand the clinical features 'at the door' so to speak, and therefore we did not mention cause of death in our study. A few papers referenced how patients with CNS scrub typhus may be classified as those with 'multi-organ failure', and therefore have 'severe' disease. None specified ARDS leading to death. We hope this answer satisfies the reviewer's point.

5. Results: In Table 1 and figure 3, the studies mentioned as Kumar 2018, Jamil 2019 and Arora 2020 are missing from the reference list.

Response:

Thank you for highlighting this and we are sorry for this mistake: Kumar 2018 is referenced as Dinesh Kumar 2018 in the reference list; Jamil 2019 is in fact Jamil 2015 in the reference list; Arora 2020 is Arora 2021 in the reference list. We have amended these all in the tables and figures in our manuscript.

Reviewer 2 comments:

1. The serological assays used (please correct in the text - indirect immunofluorescence assay, Weil-Felix test and IgM ELISA) are imperfect and accuracy highly dependent on the cut-offs used for diagnosis (if based on an acute sample only rather than paired acute and convalescent samples). The Weil-Felix test is no longer widely utilised due to lack of sensitivity and specificity. In India, where most of the included studies were from, the InBios Scrub Typhus IgM ELISA is widely used and there have been debate around an appropriate OD cut-off if based on a single acute sample. It would be helpful to allude to the imperfections of serological testing in the manuscript. Also, did some studies include molecular assays or culture? If so, was there concordance with the serological results?

Response:

Thank you for highlighting this. We agree that serological assays are imperfect, and accuracy is dependent on cut-offs. We have included this important point in the revised limitation section, and also illustrate what tests were used in our table summarising the included studies (most used InBios IgM ELISA as you highlight). As with our response to reviewer one, we have conducted sensitivity analysis with the Weil-Felix test given its low sensitivity and specificity.

One paper utilised PCR, but none cultured (assume as very few places can culture Rickettsia?). Given only one study used PCR, we did not conduct sensitivity analysis due to the low patient numbers.

2. Historically, sensori-neural hearing loss was described in patients with scrub typhus. Were there any descriptions of this in the included studies?

Response:

Thank you for raising this. No studies in our meta-analyses specified sensorineural hearing loss, and as such we elected not to include it in this review. We acknowledge this has been mentioned in literature – particularly as a primary feature in recovery (DOI: 10.1093/brain/76.1.113). We struggled to find more current references for this.

3. In the included studies, was testing of CSF samples performed? If so, it could be clinically helpful to include the cell count pattern, protein and glucose levels in scrub typhus patients with CNS involvement, even if molecular testing for Orientia tsutsugamushi is unavailable.

Response:

We agree with the reviewer that this would be extremely helpful. Among our studies, one excluded study investigated this very question (DOI: 10.4269/ajtmh.15-0119), and this only included 11 patients. We have included in our limitations and future directions section that if more studies study CSF samples in CNS scrub typhus, there is scope to properly meta-analyse findings.

4. With regards to treatment, it is important to clarify route of antibiotic administration as drug absorption through the gastrointestinal route may be impaired in critically unwell patients with organ dysfunction. There is also a discrepancy in the availability of parenteral antibiotics between regions - e.g. IV doxycycline is widely available in India but often not elsewhere. Did the included studies clarify this?

Response:

Thank you for this point. We investigated the studies and route of administration was not specified in the papers. Furthermore, we could not find specific references suggesting route of administration changes outcomes (the Cochrane review on this sadly looked at outcomes irrespective of route of administration DOI: 10.1002/14651858.CD002150.pub2). Similar to the lack of information on route of administrations in our included studies, a large review published also notes how they could not focus on therapy duration or route of administration of antibiotics owing to insufficient data (DOI: 10.1001/jamanetworkopen.2020.14487). Unfortunately, we could not reliably determine if route of administration was implicated in outcomes in the patients – though the rationale of the reviewers point may suggest this could be the case.
